# Wnt/β-catenin signaling stimulates the self-renewal of conjunctival stem cells and promotes corneal conjunctivalization

**DOI:** 10.1038/s12276-022-00823-y

**Published:** 2022-08-16

**Authors:** Esther Jang, Soomin Jin, Kyong Jin Cho, Daeseon Kim, Chang Rae Rho, Jungmook Lyu

**Affiliations:** 1 Department of Ophthalmology, Dankook University Hospital, Dankook University College of Medicine, Cheonan, Republic of Korea; 2 Department of Medical Science, Konyang University, Daejeon, Republic of Korea; 3 Department of Ophthalmology, Daejeon St. Mary’s Hospital, College of Medicine, The Catholic University of Korea, Daejeon, Republic of Korea; 4 Seoul St. Mary’s Eye Hospital, Suwon, Republic of Korea; 5 Myung-Gok Eye Research Institute, Konyang University, Daejeon, Republic of Korea

**Keywords:** Self-renewal, Stem-cell research, Experimental models of disease

## Abstract

Limbal stem cell deficiency causes conjunctivalization characterized by the covering of the corneal surface with conjunctival epithelium. However, the driving force for the encroachment of these conjunctival cells is unclear. Conjunctival stem cells are bipotent stem cells that can proliferate and differentiate into conjunctival epithelial cells and goblet cells to maintain regeneration of the conjunctival epithelium. Here, we show a robust proliferative response of conjunctival stem cells and upregulation of *Wnt2b* and *Wnt3a* gene expression in the conjunctivae of mice with induced limbal stem cell deficiency. Topical application of the Wnt/β-catenin signaling activator CHIR resulted in increased proliferation of ΔNp63α-positive stem cells in the basal layers of the bulbar and forniceal conjunctivae and enhanced invasion of conjunctival epithelial and goblet cells into the corneal surface. We also found that in cultures of stem cells isolated from the human conjunctiva, Wnt/β-catenin pathway activation improved the expansion of the ΔNp63α/ABCG2 double-positive cell population by promoting the proliferation and preventing the differentiation of these cells. These expanded stem cells formed a stratified epithelium containing goblet cells under airlift culture conditions. Our data reveal that Wnt/β-catenin signaling contributes to the pathological process of limbal stem cell deficiency by promoting the self-renewal of conjunctival stem cells and suggest that these cells are a driving force in corneal conjunctivalization.

## Ophthalmology: conjunctivalization of the cornea linked to stem cell activity

A major signaling pathway that regulates stem cell function acts as a key mediator of conjunctival invasion into the cornea following eye injuries. Using human tissue and mouse models, a team from South Korea led by Chang Rae Rho of Daejeon St. Mary’s Hospital and Jungmook Lyu of Konyang University, Daejon, showed how insults to the eye can spur the proliferation of stem cells found in the conjunctiva, the thin membrane covering the white part of the eyeball. This cell growth and self-renewal is driven by increased activity of the Wnt/β-catenin signaling pathway, leading to conjunctivalization of the cornea, the transparent outer layer of the eye, resulting in corneal opacity and loss of vision. Therapies that manipulate this signaling pathway could help improve vision for people with certain corneal diseases.

## Introduction

The cornea is a highly organized transparent tissue that covers the front portion of the eyeball. Limbal stem cells (LSCs) play a crucial role in maintaining corneal epithelial integrity, which is essential for corneal transparency^1–3^. Found in the basal epithelial layer of the limbus, a transition zone between the cornea and conjunctiva, LSCs act as a barrier to prevent the migration of the conjunctival epithelium onto the cornea^4^. Injury to the limbal epithelium, immune disorders affecting the ocular surface (such as Stevens–Johnson syndrome), and long-term contact lens wearing can result in partial or total limbal stem cell deficiency (LSCD)^5^, which leads to a process referred to as conjunctivalization. This pathological process entails encroachment of conjunctival epithelium containing goblet cells onto the corneal surface, resulting in corneal vascularization, corneal opacification, and loss of vision^6–8^. Although LSCD is characterized by conjunctivalization, the molecular and cellular mechanisms underlying this process remain largely unclear.

The conjunctival epithelium is a self-renewing tissue consisting of nonkeratinized stratified columnar cells and interspersed goblet cells. Previous studies have reported the existence of mitotically active stem cells in the bulbar and forniceal conjunctivae^9–11^. These cells have the capacity to self-renew, proliferate, and differentiate into epithelial cells and goblet cells, as has been shown in clonal culture assays^12,13^. This indicates that conjunctival stem cells (CjSCs) can constitute an ultimate source for conjunctival epithelial renewal. However, it is still unknown whether CjSCs contribute to corneal conjunctivalization.

*Wnt* signaling plays important roles in stem cell self-renewal and differentiation in adults as well as in embryonic development^14,15^. Wnt genes encode evolutionarily conserved secreted glycoproteins that activate autocrine and paracrine cell signaling pathways, including the β-catenin/TCF pathway, the planar cell polarity pathway, and the Wnt/Ca^2+^ pathway^16,17^. The canonical β-catenin/TCF pathway is activated by the binding of Wnt ligands to FZD receptors and the coreceptors LRP 5/6, resulting in inactivation of GSK-3β, which stabilizes and translocates β-catenin into the nucleus. Nuclear β-catenin acts as a transcriptional coactivator for TCF/lymphoid enhancer factor (LEF) DNA-binding transcription factors.

Several *Wnt* genes are expressed in the cornea and limbus and are upregulated after injury^18,19^. Nuclear localization of β-catenin is detectable in proliferating cells of the limbal epithelium. Moreover, activation of Wnt/β-catenin signaling by inhibition of GSK-3β drives the proliferation of LSCs^19^. Similar to the scenario during activation of Wnt/β-catenin signaling in the limbus, some conjunctival epithelial cells were reported to express β-catenin and TCF4 in the nucleus, along with CjSC markers, during in vitro expansion^20–22^. In addition, a previous study reported that deletion of Dkk2, an antagonist of Wnt/β-catenin signaling, resulted in corneal epithelial hyperplasia and invasion of conjunctival tissue into the corneal periphery in the developing ocular surface^23^. These results suggest the possibility that Wnt/β-catenin signaling acts as a key modulator of CjSCs, which promote conjunctivalization. Here, we show that LSCD causes upregulation of canonical Wnt ligand expression and the proliferative activity of CjSCs in the conjunctival epithelium. We also demonstrate that activation of Wnt/β-catenin signaling regulates CjSC self-renewal and differentiation and enhances conjunctivalization of the corneal surface.

## Materials and methods

### Human conjunctival tissue and cell culture

Human conjunctival tissues were obtained from the eye bank of Eversight (Chicago, IL, USA). The conjunctiva was retrieved from seven donors with a mean age of 65 years. This study was approved by the Institutional Review Board of Daejeon St. Mary’s Hospital (DC17TNSI0055) and was conducted in accordance with the tenets of the Declaration of Helsinki. Human conjunctival tissues were incubated in Hank’s balanced salt solution (Welgene, Gyeongsangbuk-do, Republic of Korea) containing 1% antibiotic-antimycotic (Corning, Inc., Corning, NY, USA) for 2 h at 37 °C. The conjunctival epithelium sheets were divided into several fragments of approximately 0.5–1 mm. Four or five fragments of the conjunctival epithelium were placed in 3-cm culture dishes. Primary conjunctival cells were maintained in CjSC medium (1:3 mixture of Dulbecco’s modified Eagle’s medium and Ham’s F12 medium [Welgene] supplemented with 5% fetal bovine serum [FBS; Corning, Amsterdam, The Netherlands], 1% penicillin–streptomycin [Welgene], 10 ng/mL human epidermal growth factor [Sigma, St. Louis, MO, USA], 5 µg/mL insulin [Sigma], 30 ng/mL cholera toxin [Sigma], and 500 ng/mL hydrocortisone [Sigma]). The cultures were maintained at 37 °C in an atmosphere containing 5% CO_2,_ and the culture medium was changed every other day.

For CjSC culture, conjunctival stem cells were cultured on a feeder layer. Cells were detached using trypsin-EDTA (Welgene) solution. Cells were seeded on the prepared NIH3T3 feeder layer at a density of 2.5 × 10^3^ cells/cm^2^. Cocultures were treated with 10 µg/mL Y-27632 (Tocris Bioscience, Bristol, UK) and maintained in CjSC medium. CjSCs were treated with DMSO or 3 µM CHIR99021 (Tocris Bioscience) to activate Wnt/β-catenin signaling. For differentiation, CjSCs were maintained in Roswell Park Memorial Institute (RPMI) 1640 medium supplemented with 10% FBS (Corning, Inc.) and 1% penicillin–streptomycin.

For airlift culture, CjSC colonies treated with CHIR99021 or DMSO for 5 days were isolated mechanically and dissociated into single cells by trypsin-EDTA digestion. Cells were seeded at a density of 4 × 10^4^ cells/cm^2^ in Transwell inserts coated with 0.1% gelatin. After the CjSCs were confluent, they were cultured for 14 days under airlift conditions by lowering the compartment containing the CjSC medium to the bottom of the insert.

### Mouse ocular tissue and induction of LSCD

Eight-week-old female C57BL/6 J mice were used in this study. All of the animals were treated according to the standards of the Association for Research in Vision and Ophthalmology Statement for the Use of Animals in Ophthalmic and Vision Research. For induction of LSCD, mice were anesthetized with a 1:10 dilution of a 1:1 mixture of ketamine and xylazine. For topical anesthesia, 0.5% Alcaine (Alcon, Fort Worth, TX, USA) was applied to the ocular surface. The mouse ocular surface was treated with 40% alcohol (Honeywell, Charlotte, NC, USA) using a 3 mm corneal trephine (Miltex, York, PA, USA) for 35 s. The ocular surface was irrigated with 5 mL of phosphate-buffered saline (PBS; Welgene) to wash off the remaining alcohol. The treated area of the ocular surface epithelium was removed with a spatula, and the surface was irrigated with PBS. Following wound formation, the ocular surface was treated with 300 µM CHIR or DMSO four times per day. The wound area was monitored by staining with sodium fluorescein to assess healing.

### Histological and immunofluorescence analyses

The eyes and lids of mice were removed by enucleation and immersed in 4% paraformaldehyde (PFA) overnight at 4 °C. The airlift cultures of CjSCs were fixed for 30 min with ice-cold 4% PFA. After fixation, the tissues and airlift cultures were washed, cryoprotected by immersion in 30% sucrose, and embedded in optimal cutting temperature compound. Then, they were flash-frozen in liquid nitrogen and stored at –80 °C. Cryosections were sliced at a thickness of 10 μm with a cryostat (Leica, Wetzlar, Germany) and mounted on glass slides. The sections were thawed and postfixed with 4% PFA for 10 min. For histological assessment, sections were permeabilized with 0.5% Triton X-100 and stained with PAS (Sigma) and Alcian blue (Abcam) staining kits according to the manufacturer's instructions. Images were acquired using a microscope (Leica).

For immunostaining, tissue and airlift culture sections were washed with PBS and incubated for 1 h with 2% bovine serum albumin (Gibco, Dublin, Ireland) and 5% normal goat serum (Jackson ImmunoResearch, West Grove, PA, USA) diluted in PBS. The sections were sequentially incubated overnight at 4 °C with primary antibodies against rabbit K13 (Abcam), rabbit K7 (Abcam), rabbit ΔNp63α (BioLegend, San Diego, CA, USA), mouse ABCG2 (Santa Cruz Biotechnology, Dallas, Tx, USA), rabbit PAX6 (BioLegend), mouse Cyclin D1 (Abcam), rabbit β-catenin (Sigma), rabbit active β-catenin (Cell Signaling Technology, Danvers, MA, USA), and mouse Ki67 (Beckman Coulter, Brea, CA, USA). After washing with PBS, the sections were incubated for 1 h at room temperature with Alexa Fluor 488- or Cy3-conjugated secondary antibodies (Thermo Fisher Scientific, Waltham, MA, USA) and counterstained with Hoechst 33258 (Sigma). Images were acquired using a fluorescence microscope with an Axiocam camera (Zeiss, Jena, Germany). Cells grown on coverslips were fixed with ice-cold 4% PFA for 15 min at room temperature, permeabilized with 0.2% Triton X-100, and immunostained as described above. The number of antibody-labeled cells was quantified in eight randomly selected fields per section or coverslip. Data obtained from at least three independent experiments were averaged and are presented as the mean ± S.D. values. Two-tailed Student’s *t* test was used to compare two experimental groups.

### BrdU and EdU staining

CjSC proliferation was assessed by injecting 1 mg of BrdU (Sigma) in 200 µL of PBS (Welgene) after LSC removal or on the day after wounding. Tissue sections were postfixed with 4% PFA, permeabilized, and incubated with an anti-ΔNp63α antibody at 4 °C overnight. Then, the sections were incubated in 1 M HCl for 30 min at 37 °C and washed three times for 10 min each with 0.1 mM borate buffer. After the tissue sections were washed with PBS, a rat anti-BrdU antibody (Abcam) was applied and incubated overnight at 4 °C. Then, the slides were incubated with the appropriate secondary antibody for 1 h at room temperature.

For double immunostaining of EdU and ABCG2, cells grown on coverslips were treated with 5 µM EdU (Thermo Fisher Scientific) 6 h before fixation. Then, the cells were fixed with 4% PFA, permeabilized with 0.2% Triton X-100, and subjected to immunostaining with an anti-ABCG2 antibody, as described above. To detect EdU, Click-iT EdU Imaging Kits (Thermo Fisher Scientific) were used according to the manufacturer’s instructions.

### RNA extraction, RT–PCR, and quantitative real-time PCR

Total RNA was isolated from conjunctival cells and conjunctival tissues using TRIzol reagent (Invitrogen, Carlsbad, CA, USA) according to the manufacturer’s instructions. cDNA was synthesized from RNA by reverse transcription with a Superscript III Kit (Invitrogen). PCR amplification was carried out using specific primer pairs and an nTaq-HOT Kit (Enzynomics, Republic of Korea). Quantitative real-time PCR was performed using SmartGene^TM^ Q-PCR Master Mix (SmartGene, Republic of Korea), and expression in the samples was quantified by amplifying *GAPDH* as the internal control for each sample. All experiments were performed at least three independent times.

## Results

### LSCD causes the proliferation of CjSCs

**Fig. 1:**
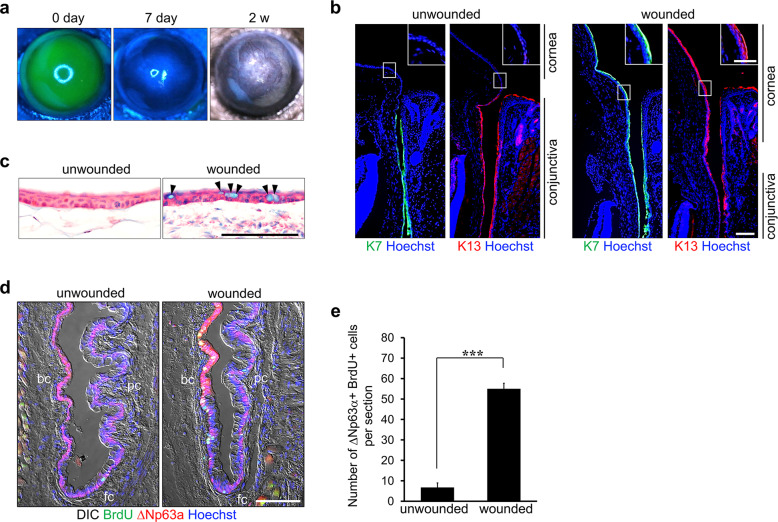
Induction of LSCD causes corneal conjunctivalization and increases the proliferative activity of CjSCs. **a** Induction of LSCD in C57BL/6 mice using complete corneal and limbal epithelial debridement. The corneas were stained with sodium fluorescein immediately (0 days) and 7 days after epithelial debridement. The defect was completely resurfaced at 7 days. Light microscopy 2 weeks post-wounding showed blood vessels penetrating into the cornea and severe corneal opacity. **b**, **c** The wound inflicted by epithelial debridement resulted in corneal conjunctivalization. Sagittal sections from unwounded and wounded ocular surface tissues were immunostained with antibodies against the conjunctival epithelial cell markers K7 and K13 (**b**). Hoechst dye was used as a counterstain. K7 and K13 expression were detectable at the corneal surface on Day 7 post-wounding. Goblet cells appeared at the periphery of the resurfaced cornea 7 days after wounding (**c**). Sagittal sections from unwounded and wounded ocular surface tissues were stained with Alcian blue. The sections were counterstained with hematoxylin and eosin. The arrowheads indicate Alcian blue-positive goblet cells. **d** The proliferative activity of CjSCs was increased in the bulbar and forniceal conjunctivae of mice with LSCD. BrdU was applied on Day 2 post-wounding. On Day 3 after wounding, ocular surface tissue sections were immunostained with antibodies against BrdU and ΔNp63α (a putative marker of CjSCs). **e** Quantitative analysis of ΔNp63α/BrdU double-positive CjSCs in the conjunctivae of mice with LSCD (wounded) and on a normal ocular surface (unwounded). The error bars indicate the mean ± S.D. of three independent experiments. ***P < 0.001. Scale bars, 100 µm; inset, 50 µm. bc bulbar conjunctiva, fc forniceal conjunctiva, pc palpebral conjunctiva.

We used chemical and mechanical epithelial debridement to induce LSCD in mice. Removal of the entire corneal and limbal epithelia was confirmed by fluorescein staining (Fig. 1a). On Day 7 after epithelial debridement, fluorescein staining was not observed, indicating that the wound area was almost completely resurfaced. Corneal opacity was also observed with vessel formation after 2 weeks. On Day 7 post wounding, immunostaining for K7 and K13 (markers of conjunctival epithelial cells) revealed the expression of these proteins in the suprabasal and superficial cell layers of the corneal surface as well as in the conjunctival epithelium (Fig. 1b)^24,25^. Moreover, Alcian blue-positive goblet cells were detected in the corneal periphery (Fig. 1c and Supplementary Fig. 1). These results indicated LSCD-like pathological features such as corneal conjunctivalization and neovascularization.

To determine whether CjSCs are involved in conjunctivalization, we injected BrdU intraperitoneally into mice following epithelial debridement and performed immunostaining on ocular tissue sections with anti-ΔNp63α and anti-BrdU antibodies to identify CjSCs. On Day 3 post-wounding, we observed ΔNp63α/BrdU double-positive cells predominantly in the basal layer of the bulbar and forniceal conjunctival epithelia (Fig. 1d). Quantitative analysis showed that epithelial debridement markedly increased the number of these CjSCs compared to that in the unwounded ocular surface (from 6.8 ± 2.2 cells to 55.0 ± 2.7 cells per section in the bulbar and forniceal conjunctivae) (Fig. 1e). These results suggest that LSCD increases the proliferative activity of CjSCs.

### Wnt/β-catenin signaling promotes the proliferation of CjSCs and conjunctivalization

**Fig. 2:**
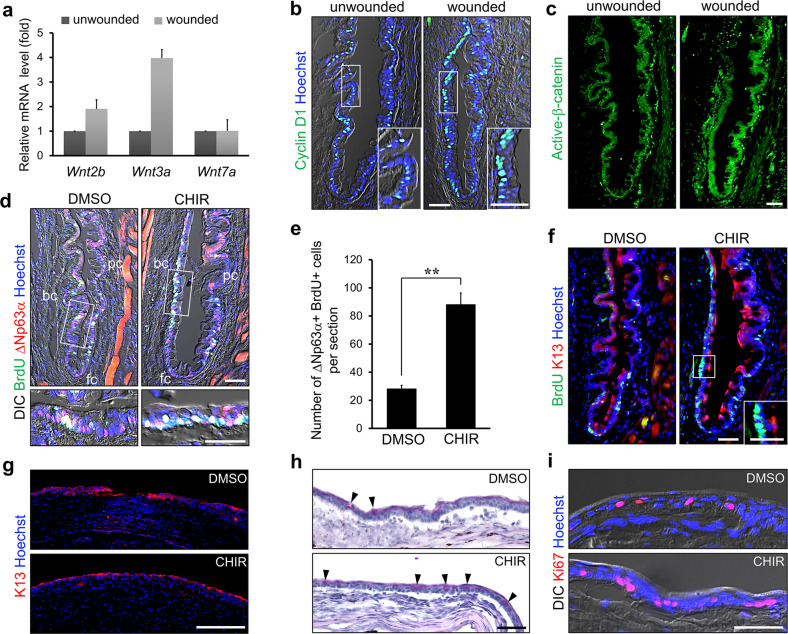
Wnt/β-catenin signaling is activated and promotes CjSCs proliferation and corneal conjunctivalization in mice with LSCD. **a** Quantitative real-time PCR analysis of *Wnt* gene expression in the conjunctivae of mice with a wounded or unwounded ocular surface is shown. **b**, **c** Three days post-wounding, ocular surface tissue sections were immunostained with anti-Cyclin D1 (**b**) and anti-active β-catenin (**c**) antibodies and counterstained with Hoechst dye. The number of cyclin D1-positive cells and the fluorescence intensity of active β-catenin were increased in the conjunctivae of mice with wounded ocular surfaces compared with that of mice with unwounded ocular surfaces. **d** Treatment of the ocular surface with the GSK-3β inhibitor CHIR99021 (CHIR) resulted in increased proliferation of CjSCs. Ocular surface tissues treated with CHIR or DMSO as a control for 2 days after wounding were immunostained with anti-BrdU and anti-ΔNp63α antibodies following injection of BrdU on the first-day post-wounding. **e** Quantitative analysis of ΔNp63α/BrdU double-positive CjSCs in the bulbar and forniceal conjunctivae of ocular surface tissues treated with CHIR or DMSO is shown. CHIR treatment significantly increased the number of BrdU/ΔNp63α double-positive cells. The error bars indicate the mean ± S.D. of four independent experiments. **f** Ocular surface tissue sections were immunostained with anti-BrdU and anti-K13 antibodies. BrdU-positive cells were predominantly detected in cells in the basal layer of the conjunctiva but not in superficial epithelial cells expressing K13. **g**–**i** Seven days post-wounding, ocular surface tissues treated with CHIR or DMSO were immunostained with anti-K13 or anti-Ki67 (a marker of proliferating cells) antibodies and stained with PAS (a goblet cell marker). Immunostaining for K13 in the central corneal sections revealed more complete resurfacing with conjunctival epithelial cells in CHIR-treated ocular surface tissues (**g**). CHIR treatment increased the numbers of PAS-positive goblet cells (arrowheads in **h**) and Ki67-positive proliferating cells (**i**) in the resurfaced epithelium of the peripheral cornea. **P < 0.01. Scale bars, 50 µm in **b**–**d**, **f**, **h**, and **i**; 200 µm in **g**; inset, 20 µm in **f**. bc bulbar conjunctiva, fc forniceal conjunctiva, pc palpebral conjunctiva.

To investigate whether Wnt signaling is implicated in conjunctivalization, we first examined the expression of Wnt ligands in the conjunctival epithelium following epithelial debridement. Quantitative real-time PCR revealed that *Wnt2b* and *Wnt3a* expression was significantly higher 7 days post-wounding compared to that in the unwounded ocular surface (Fig. 2a). Wnt2b and Wnt3a are Wnt ligands that activate β-catenin-dependent signaling. Immunostaining for Cyclin D1, a Wnt target gene, revealed that the number of Cyclin D1-positive cells was increased in the wounded conjunctival epithelium 3 days post-wounding (Fig. 2b). In addition, the immunostaining intensity and the level of active β-catenin were increased in the wounded mice (Fig. 2c and Supplementary Fig. 2a). These results demonstrate that epithelial injury in the cornea and limbus results in activation of Wnt/β-catenin signaling in the conjunctival epithelium.

Next, we tested whether activation of Wnt/β-catenin signaling increases the proliferative activity of CjSCs in mice with LSCD. To this end, ocular surfaces were treated with the GSK-3β inhibitor CHIR99021 (CHIR) via eye drops for 2 days after epithelial debridement and were then subjected to immunostaining for BrdU and ΔNp63α following injection of BrdU on Day 1 post-wounding. CHIR treatment markedly increased the number of BrdU/ΔNp63α double-positive cells in the bulbar and forniceal conjunctivae (Fig. 2d). Quantitative analysis showed a significant increase in the number of these CjSCs after CHIR treatment compared to that after DMSO (control) treatment (from 23.3 ± 2.3 cells to 88.3 ± 8.0 cells per section) (Fig. 2e). Immunostaining of these tissues for BrdU and K13 revealed that BrdU was not detected in most cells expressing K13 in the CHIR-treated conjunctiva (Fig. 2f). This finding indicates that CHIR treatment did not affect the proliferation of superficial epithelial cells in the bulbar and forniceal conjunctivae. In addition, a significant decrease in the number of BrdU/ΔNp63α double-positive cells (from 21.8 ± 4.7 cells to 4.2 ± 2.2 cells per section) was observed in the bulbar and forniceal conjunctivae after treatment of the ocular surface with recombinant Dickkopf1 (DKK1) for 2 days post-wounding, to inhibit Wnt/β-catenin signaling (Supplementary Fig. 2b).

To further assess conjunctivalization, we clinically evaluated corneal neovascularization and opacity in ocular surfaces treated with CHIR or DMSO for 7 days post-wounding. CHIR treatment resulted in a significant increase in the corneal neovascularization score (from 8.2 ± 1.0 to 11.0 ± 0.7), although no significant difference in the corneal opacity score was observed between ocular surfaces treated with CHIR and DMSO (Supplementary Fig. 3a, b). We next performed immunostaining with an anti-K13 antibody and periodic acid–Schiff (PAS) staining 7 days after treatment with CHIR or DMSO. Immunostaining of corneal sections showed that compared to control treatment, CHIR treatment resulted in a more regular corneal surface with an increased K13-positive cell population (Fig. 2g and Supplementary Fig. 3c). Moreover, CHIR treatment increased the number of PAS-positive cells in the peripheral cornea compared to that observed after DMSO treatment (Fig. 2h and Supplementary Fig. 3d). Interestingly, proliferative cells were observed in the basal layer of the resurfaced epithelium. The number of proliferative cells was increased in CHIR-treated corneal sections compared to DMSO-treated corneal sections, as shown by immunostaining of for the cell cycle marker Ki67 in corneal sections (Fig. 2i and Supplementary Fig. 3e). In addition, taken together, our data suggest that activation of Wnt/β-catenin signaling promotes CjSC proliferation and corneal conjunctivalization.

### Wnt/β-catenin signaling promotes the self-renewal of CjSCs

**Fig. 3:**
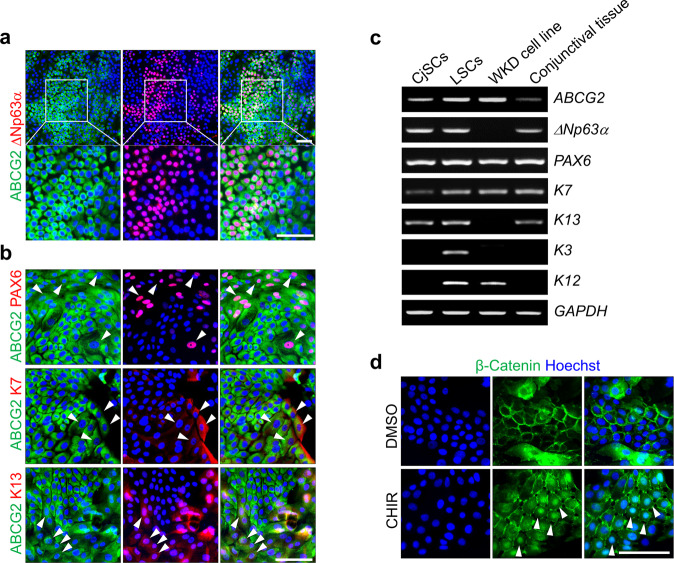
CjSC cultures from the human conjunctiva. **a**, **b** Immunostaining of CjSC cultures with antibodies against the stem cell markers ABCG2 and ΔNp63α (**a**) and the epithelial markers PAX6, K7, and K13 (**b**) is shown. Cells isolated from the human conjunctiva were cultured on a layer of mitotically inactivated feeder cells for 10 days. Hoechst dye was used as a counterstain. ABCG2/ΔNp63α double-positive cells were seen in cell colonies. Interestingly, cells expressing low levels of ABCG2 did not express ΔNp63α. These cells expressed PAX6, K7, and K13. The arrowheads indicate cells with low ABCG2 expression. **c** RT–PCR analysis of CjSC and LSC cultures, the WKD conjunctival cell line, and conjunctival tissues confirmed the expression of CjSC and conjunctival epithelial markers and the absence of the corneal epithelial markers K3 and K12. **d** CHIR activates Wnt/β-catenin signaling in CjSC cultures. CjSC cultures were treated with 3 µM CHIR or DMSO for 2 days and immunostained with an anti-β-catenin antibody. β-catenin was strongly expressed in the nucleus (arrowheads) in CHIR-treated cultures but was localized to the cell membrane in DMSO-treated cultures. Scale bars, 100 µm.

Self-renewal enables CjSCs to proliferate while retaining bipotency^12^. To examine whether Wnt/β-catenin signaling regulates the self-renewal of CjSCs, we established an in vitro culture system using CjSCs isolated from the human conjunctiva. Immunostaining of cultured CjSCs for CjSC markers revealed ABCG2-positive cell clusters in colonies and differential expression levels of ABCG2 among cells. ABCG2 was strongly expressed in ΔNp63α-positive cells, whereas its expression was decreased in ΔNp63α-negative cells (Fig. 3a). Interestingly, cells with low levels of ABCG2 expressed Pax6 (Fig. 3b, arrowheads in the upper panels), which is expressed in the fully differentiated, stratified ocular surface epithelium of the adult eye^26^. Thus, Pax6 expression in these cultures indicates spontaneous differentiation of CjSCs. Along the margins of ABCG2-positive cell clusters, we further confirmed CjSC differentiation by the presence of ABCG2-low cells undergoing morphological changes as well as expressing K7 and K13 (Fig. 3b, arrowheads in middle and lower panels). Semiquantitative RT–PCR analyses of cultures further confirmed the expression of ocular surface stem cell and epithelial cell markers comparable to that in LSC cultures and human conjunctival tissue (Fig. 3c). In addition, in these cultures, the majority of cells showed predominant membrane localization of β-catenin. When the cultures were treated with CHIR, β-catenin localization was markedly increased in the nucleus as well as the cytoplasm; in contrast, the membrane localization of β-catenin was decreased (Fig. 3d).

**Fig. 4:**
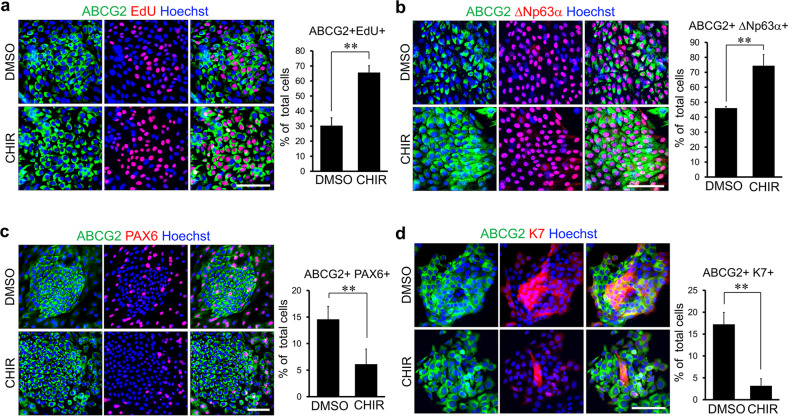
CHIR-mediated Wnt/β-catenin signaling activation increases the self-renewal of CjSCs. Immunostaining analysis of ABCG2 and EdU (**a**), ΔNp63α (**b**), PAX6 (**c**), and K7 (**d**) in CjSC cultures treated with CHIR or DMSO for 5 days. Nuclei were counterstained with Hoechst dye. The quantitative analysis results are shown in the left panel. CHIR-treated CjSC cultures showed a significant increase in the numbers of ABCG2/EdU (**a**) and ABCG2/ΔNp63α (**b**) double-positive cells compared with those in DMSO-treated cultures, whereas the numbers of ABCG2/PAX6 (**c**) and ABCG2/PAX6 (**d**) double-positive cells were decreased. The data are shown as the mean ± S.D. of four independent experiments. **P < 0.01. Scale bars, 100 µm.

Next, we tested the effects of Wnt/β-catenin signaling on CjSC proliferation and differentiation. CjSC cultures pretreated with CHIR or DMSO were immunostained with the anti-ABCG2 antibody following 5-ethynyl-2’-deoxyuridine (EdU) staining alone or in combination with the anti-ΔNp63α antibody. The majority of EdU-positive cells expressed a high level of ABCG2, indicative of the active proliferation of CjSCs (Fig. 4a). The number of proliferative CjSCs was increased in cultures treated with CHIR compared to those treated with DMSO (from 30.2 ± 5.3% to 65.5 ± 4.6% for the percentage of ABCG2/EdU double-positive cells among total cells). Compared to DMSO treatment, CHIR treatment also significantly increased the number of ABCG2/ΔNp63α double-positive CjSCs (from 46.1 ± 1.1% to 74.4 ± 7.4% of total cells) (Fig. 4b). In contrast, the number of differentiating cells was significantly decreased in the cultures treated with CHIR compared to those treated with DMSO, as determined by quantitative analysis of immunostaining for epithelial markers (from 44.0 ± 3.2% to 26.5 ± 2.7% and from 17.2 ± 2.7% to 3.1 ± 1.6% for the percentages of ABCG2/Pax6 and ABCG2/K7 double-positive cells among total cells, respectively) (Fig. 4c, d). These results demonstrate that activation of Wnt/β-catenin signaling promotes the self-renewal of CjSCs and inhibits their spontaneous differentiation.

### Activation of Wnt/β-catenin signaling inhibits CjSC differentiation

**Fig. 5:**
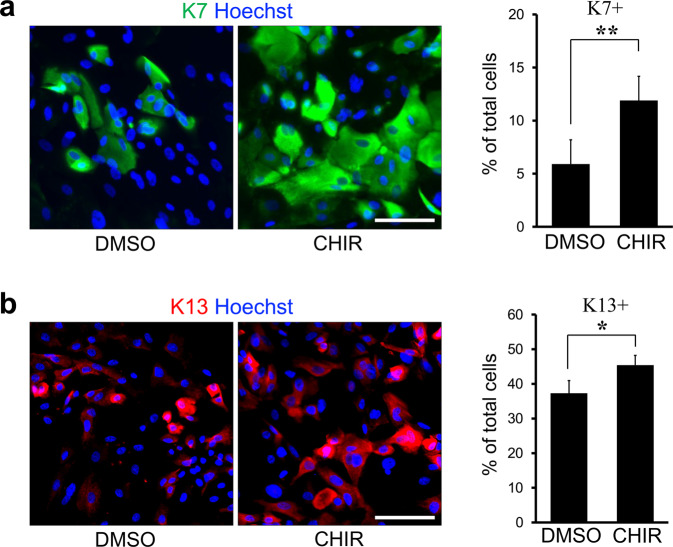
Pretreatment of CjSCs with CHIR increases the differentiated cell population. CjSC cultures were treated with CHIR or DMSO for 5 days and were then grown under differentiation conditions without CHIR for 21 days. The cultures were immunostained with anti-K7 (**a**) and anti-K13 (**b**) antibodies. Nuclei were counterstained with Hoechst dye. Quantitative analysis of K7- and K13-positive cells showed a significant increase in the differentiated cell population in CjSC cultures pretreated with CHIR compared to those pretreated with DMSO. The data are shown as the mean ± S.D. of three independent experiments. *P < 0.05; **P < 0.01. Scale bars, 50 µm.

**Fig. 6:**
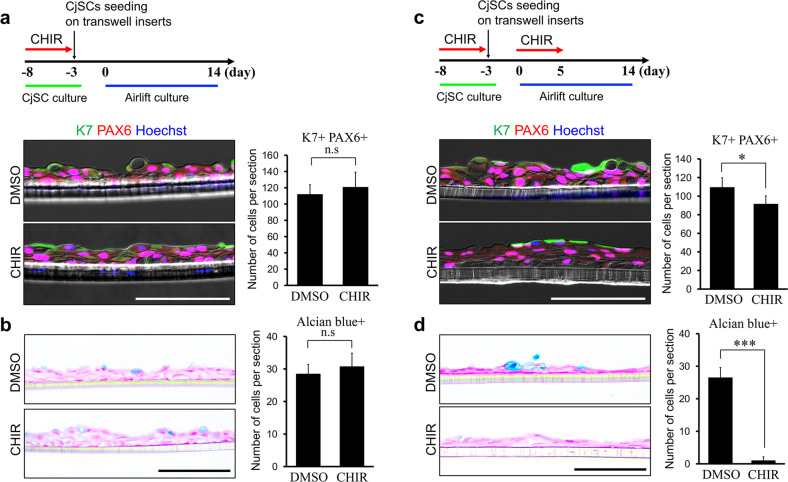
Wnt/β-catenin signaling inhibits the differentiation of CjSCs in airlift culture. **a**, **b** CjSCs were grown in the presence of CHIR or DMSO for 5 days, seeded on Transwell inserts, and cultured under airlift conditions for 14 days. **c**, **d** CjSCs grown with CHIR were cultured under airlift conditions in the presence of CHIR or DMSO. Immunostaining for K7 and PAX6 in the cultivated cell sheets is shown in **a** and **c**, respectively. Goblet cells revealed by Alcian blue staining are shown in **b** and **d**. Quantitative analyses of K7/PAX6 double-positive differentiating cells and Alcian blue-positive goblet cells are shown in the left panels. Notably, the numbers of differentiating cells and goblet cells were significantly decreased in CHIR-treated airlift cultures compared with DMSO-treated cultures. The data are shown as the mean ± S.D. of three independent experiments. *P < 0.05; ***P < 0.001; n.s. nonsignificant. Scale bars, 100 µm.

Next, we assessed the differentiation capacity of cells treated with CHIR to determine their potency as CjSCs. CjSCs were grown with CHIR or DMSO for 5 days and were then cultured under differentiation conditions in the absence of CHIR for 21 days. As expected, the numbers of K7- and K13-positive cells were significantly higher in cultures pretreated with CHIR than in control cultures (Fig. 5a, b). This increase in the number of differentiated cells may be a result of an increase in the CjSC population. To rule out this possibility, CjSCs isolated from cultures treated with CHIR or DMSO were cultured under airlift conditions, which produce a well-differentiated and stratified epithelium. On Day 14, airlift cultures showed stratification of Pax6-positive cells into two to three layers in which K7 was expressed along the apical surface (Fig. 6a). Moreover, Alcian blue staining showed goblet cells in the surface cell layer (Fig. 6b). However, no marked differences were evident between the airlift cultures of CjSCs pretreated with CHIR and DMSO in terms of the Pax6-positive cell layer, K7 protein expression, and numbers of K7/Pax6 double-positive differentiating cells and Alcian blue-positive goblet cells. To further evaluate the role of Wnt/β-catenin signaling in the differentiation of CjSCs, CjSCs were grown under airlift conditions with CHIR or DMSO treatment for 5 days. Although normal epithelial differentiation was observed under both conditions, with K7 expression, the K7/Pax6 double-positive cell population was reduced in airlift cultures treated with CHIR compared to those treated with DMSO (Fig. 6c). Moreover, in airlift cultures treated with CHIR, the number of goblet cells was significantly decreased compared to that in cultures treated with DMSO (Fig. 6d). Taken together, these results indicate that Wnt/β-catenin signaling activation inhibits CjSC differentiation and increases the expansion of CjSCs with stem cell potency.

## Discussion

Conjunctivalization is a pathological process of LSCD and results in loss of corneal clarity^5^. This process is characterized by the covering of the corneal surface with conjunctival epithelial cells and goblet cells; however, the driving force that promotes the invasion of these conjunctival cells is unclear. The adult conjunctiva contains a population of quiescent or slow-cycling somatic stem cells^9,12,27^. In this study, we showed a robust proliferative response of CjSCs in the bulbar and forniceal conjunctivae following epithelial debridement that induces LSCD. Basal mitotic stem cells of the conjunctiva migrate to the surface and become quiescent, terminally differentiated cells but do not undergo lateral migration during normal tissue homeostasis^9^. In the process of conjunctivalization, however, CjSCs can be activated to proliferate, forcing CjSCs and/or their progeny to migrate into the cornea. This hypothesis is supported by the observations that the BrdU-positive cell distribution extended toward the cornea in the conjunctiva 3 days post wounding, and that 7 days post-wounding, Ki67-positive proliferating cells were present in the basal layer of corneal epithelium newly resurfaced by conjunctival cells. Previous studies have found ΔNp63α-positive cells with proliferative potential in the basal layer of pterygia, abnormalities characterized by invasion of a conjunctivalized, fibrovascular pannus^28–30^. These findings are not surprising, because this process resembles the early stage of corneal wound healing. Following corneal wounding, the proliferation of LSCs or transit-amplifying cells located in the limbus is initially boosted, enticing new basal epithelial cells to migrate centripetally into the wound area^2,3^.

Our study indicates that Wnt/β-catenin signaling drives the self-renewal of CjSCs in the process of conjunctivalization. Extrinsic signals and intrinsic factors regulate stem cell self-renewal. The transcription factor TCF4 is coexpressed with ΔNp63α in basal cells of the mouse conjunctival epithelium; TCF4 thus has a potential role in CjSC proliferation. TCF4 binds to β-catenin to drive the transcription of Wnt target genes associated with proliferation, such as *Cyclin D1*^31^. We observed upregulation of the *Wnt2b* and *Wnt3a* genes and increases in the protein levels of active β-catenin and Cyclin D1 in the conjunctival epithelium after wounding. These data indicate that the endogenous β-catenin/TCF4 pathway can be activated in CjSCs in response to corneal and limbal epithelial injury to stimulate their proliferation. CHIR treatment, which leads to activation of the β-catenin/TCF pathway^32,33^, enhanced the proliferation of CjSCs in both conjunctival tissues and in vitro culture. The ability of self-renewal division is a unique property of stem cells that allows them to maintain their functions^34,35^. Thus, CjSC self-renewal can be defined as the proliferation of bipotent cells giving rise to both conjunctival epithelial cells and goblet cells. Importantly, we found that in vitro culture, CjSCs retain differentiation potential when grown with CHIR, whereas their spontaneous differentiation is inhibited.

We showed that the cornea was resurfaced rapidly by conjunctival epithelial cells, with an increased population of goblet cells and neovascularization, when the mouse ocular surface was treated with CHIR. Although we propose that the increased CjSC population may result in the generation of new conjunctival cells during conjunctivalization, it is unclear how the production of goblet cells occurs given the activation of Wnt signaling, which inhibits goblet cell differentiation. One possible explanation is that other factors function to allow the differentiating goblet cells to overcome or bypass inhibition by Wnt/β-catenin signaling. In the ocular surface of adult mice, conditional inhibition of Notch signaling causes conjunctival epithelial hyperplasia and complete suppression of goblet cell differentiation^36,37^. Interaction/crosstalk of the Wnt and Notch signaling pathways in development and disease has been well documented^38^. Previous studies have also suggested that Notch can downregulate Wnt signaling by promoting the degradation of β-catenin^39^. Future studies should investigate the relationship between Notch and Wnt signaling in goblet cell differentiation.

Based on our findings, we propose that CjSCs have pathologic potential that can be stimulated by aberrant activation of Wnt/β-catenin signaling and in response to epithelial injury that induces LSCD. Furthermore, our findings suggest that modulation of Wnt/β-catenin signaling is a possible therapeutic strategy for pterygia and LSCD.

## Supplementary information

Supplementary information
