# Supplementary material for: Wnt/β-catenin signaling stimulates the self-renewal of conjunctival stem cells and promotes corneal conjunctivalization
Source: Exp Mol Med. 2022 Aug 16;54(8):1156–64. doi: 10.1038/s12276-022-00823-y (PMC9440202; doi:10.1038/s12276-022-00823-y)
Supplement: Supplementary file 1 — Supplementary information [file 12276_2022_823_MOESM1_ESM.pdf]

Supplementary Information

Supplementary Material and Methods

Clinical assessment of corneal opacity and neovascularization

The cornea was observed using a slit-lamp or stereo microscopes on day 7 after wounding. As previously described [1], the degree of corneal opacity was scored using on a numerical scale from 0 to 4 as follows: 0, transparent cornea; 1, mild opacity; 2, moderate opacity with discernible iris features; 3, dense opacity with loss of defined iris detail except pupil margins; 4, severe opacity with no posterior view. The degree of corneal neovascularization was rated on a scale from 0 to 3 per corneal quadrant, using a grid system based on the centripetal invasion of neovascular branches from the limbus, as previously described [2]. The scores for each quadrant were then summed to obtain the neovascularization index (range, from 0 to 12) for each eye.

Western Blotting

The epithelium of the bulbar and forniceal conjunctiva was mechanically scraped and lysed in a lysis buffer (25 mM Tris-HCl (pH 7.4), 150 mM sodium chloride, 5 mM EDTA, 1× Triton X-100, 10% glycerol, 10 mM sodium pyrophosphate, 10 mM β-glycerophospate, 1 mM sodium orthovanadate, 1× protease inhibitor, 10 mM sodium fluoride, 1 mM phenylmethylsulfonyl fluoride). Equal amounts of protein were separated by 10% SDS-PAGE and transferred onto PVDF membranes. After blocking with 5% non-fat dry milk or 5% BSA in TBS with 0.02% Tween-20 for 1 h, the blots were incubated with anti-active β-catenin (Cell Signaling Technology) and anti-β-catenin (Sigma) antibodies. The membranes were washed with TBST three times for 5 min time and then incubated with horseradish peroxidase secondary antibody (Thermo Fisher Scientific) for 1 h 30 min at room temperature. The bound secondary antibody was then detected using Clarity™ Western ECL Substrate (Bio-Rad Laboratories Inc, Hercules, CA, USA) and the ChemiDoc Touch Imaging System (Bio-Rad).

Supplemental References

1. Keadle, T. L. & Stuart, P. M. Interleukin-10 (IL-10) ameliorates corneal disease in a mouse model of recurrent herpetic keratitis. *Microb. Pathog*. **38**, 13–21 (2005).
2. Dana, M. R. & Streilein, J. W. Loss and restoration of immune privilege in eyes with corneal neovascularization. *Investig. Ophthalmol. Vis. Sci.* **37**, 2485–2494 (1996).

Supplementary Figures and Legends

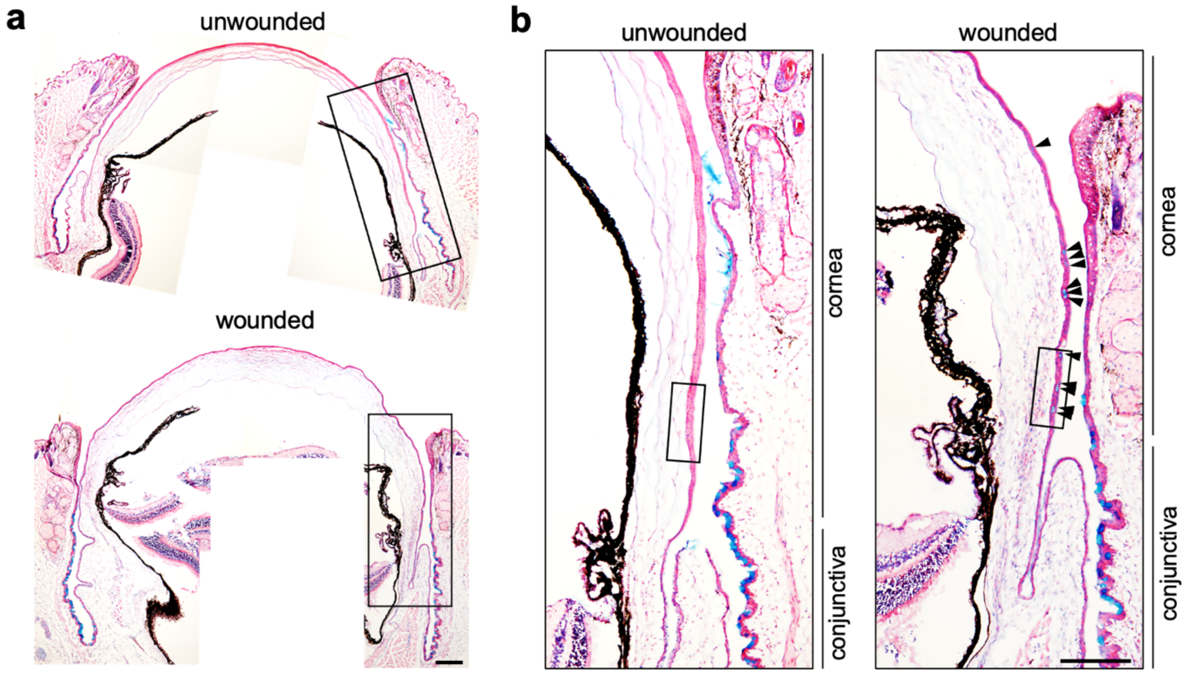

Supplementary Fig. 1. The induction of LSCD causes coverage of the cornea by conjunctival epithelium, replete to goblet cells. (a) Sagittal sections from eyes with LSCD (wounded) and a normal ocular surface (unwounded), stained with Alcian blue. (b) Higher magnification of insets (boxed areas in a) reveals the appearance of Alcian blue-positive goblet cells (arrowheads) at the periphery of the resurfaced cornea after wounding. Boxed insets are shown in Fig. 1c at higher magnification. Scale bars, 200 µm.

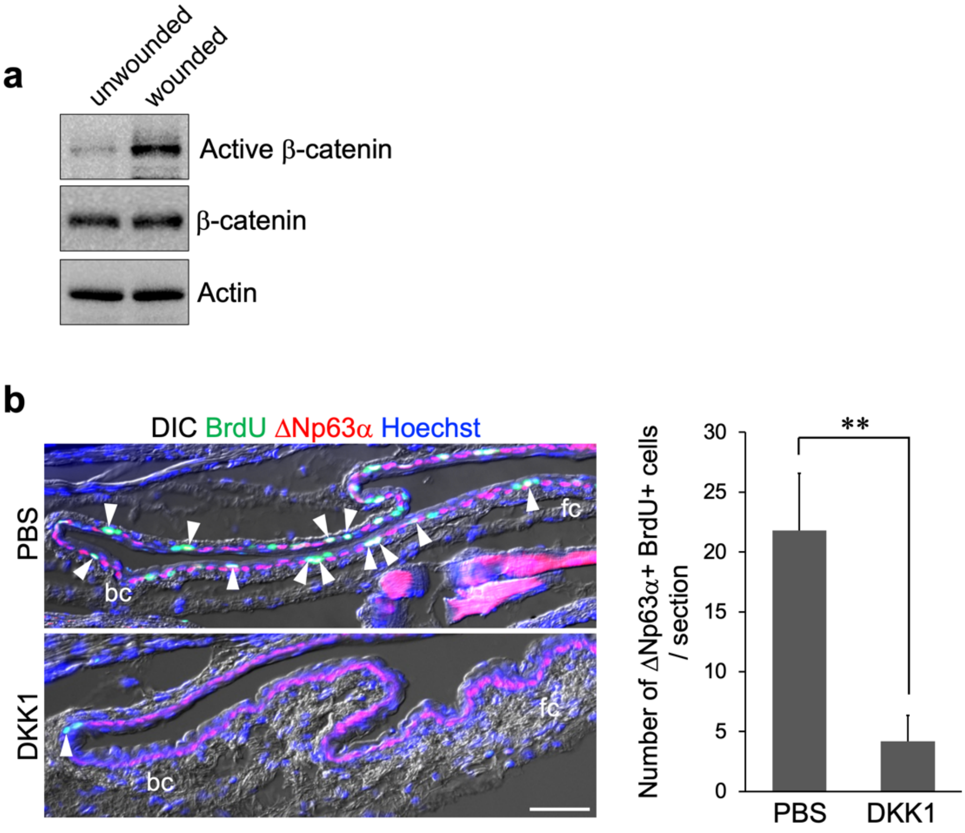

Supplementary Fig. 2. Wnt/β-catenin signaling is activated in the conjunctival epithelium of mice with LSCD, and blockage of its signaling decreases the proliferation of CjSCs. (a) Western blot analysis of lysates from the conjunctival epithelium of mice with unwounded or wounded ocular surfaces. The expression of active β-catenin was markedly increased at 3 days pos-wounding as compared to that in unwounded conjunctival epithelium. (b) DKK1-mediated inhibition of Wnt/β-catenin signaling results in decreased proliferation of CjSCs. Ocular surface tissues were treated with DKK1 or PBS for 2 days after wounding and subjected to immunostaining for BrdU and -ΔNp63α following the injection of BrdU at day 1 post-wounding. Arrowheads indicate BrdU/ΔNp63α double-positive CjSCs in the bulbar and forniceal area of conjunctiva. Quantitative analysis shows a significant decrease in the number of BrdU/ΔNp63α double-positive cells in DKK1-treated conjunctival epithelium compared to that in PBS-treated counterparts. Data are shown as the mean ± S.D. of three independent experiments. $^{**}P < 0.01$. Scale bar, 50 µm. bc, bulbar conjunctiva; fc, forniceal conjunctiva.

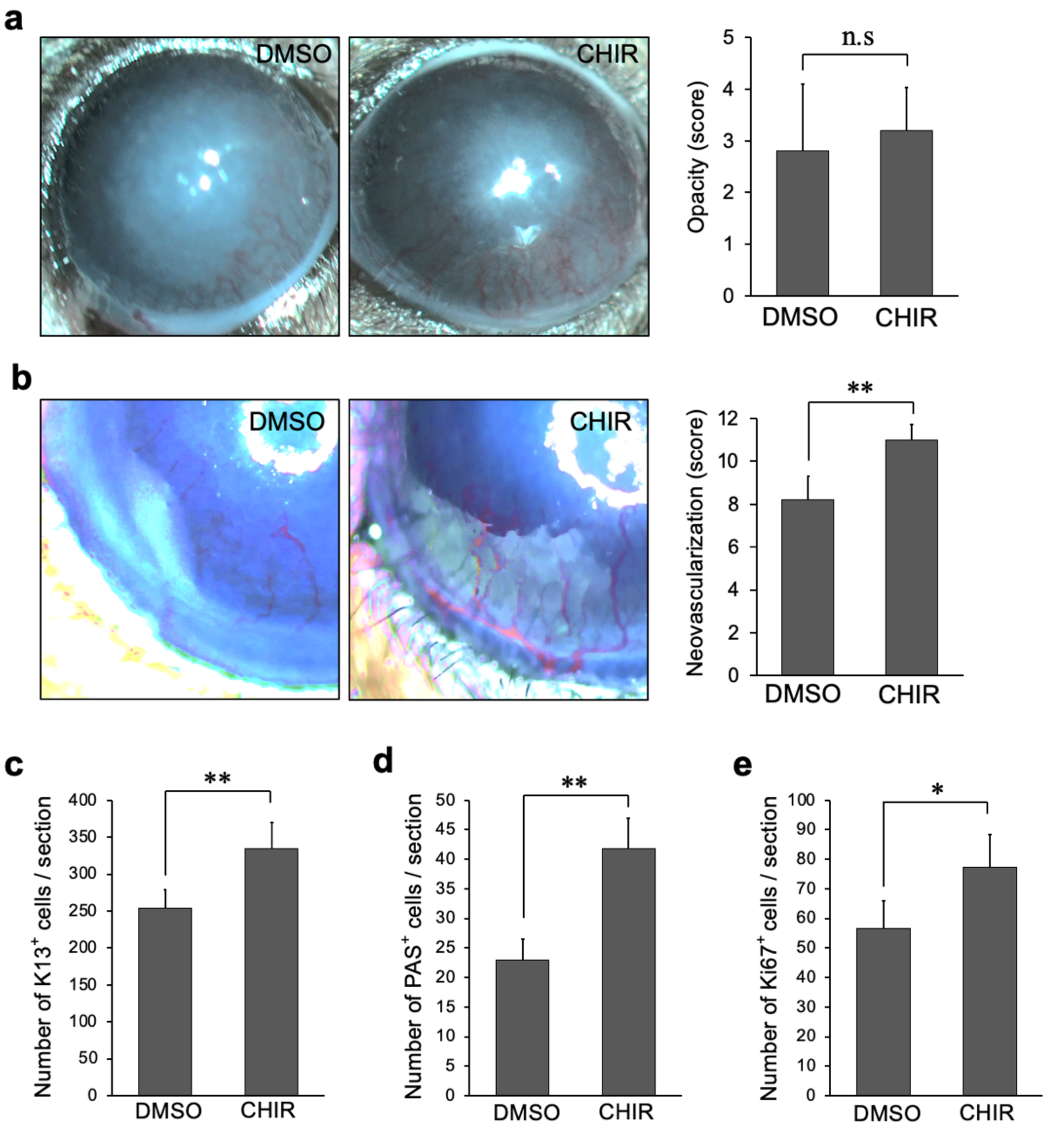

Supplementary Fig. 3. CHIR treatment enhances conjunctivalization of the corneal surface. (a, b) Clinical assessment of corneal opacity and neovascularization. Corneal opacity (a) and neovascularization (b) were scored and photographed in the ocular surfaces treated with CHIR or DMSO for 7 days post-wounding. Corneal opacity is observed in all eyes after wounding (left panels in a). However, no significant difference is seen in the corneal opacity score between CHIR- and DMSO-treated eyes (right panel in a). The neovessels growing from limbal vessels toward the central cornea are also observed in the cornea after wounding (left panel in b). Quantitative analysis of the corneal neovascularization shows a significantly higher score in the CHIR-treated ocular surfaces than in the PBS-treated counterparts (right panel in b). (c) Quantitative analysis of K13-

positive cells in the superficial layer of the resurfaced epithelium of the central cornea shown in Fig. 2g. (d, e) Quantitative analysis of PAS- and Ki67-positive cells in the resurfaced epithelium of the peripheral cornea shown in Fig. 2h and i. The number of K13-positive conjunctival epithelial cells, PAS-positive goblet cells, and Ki67-positive proliferating cells were significantly increased in the CHIR-treated ocular surface as compared to the DMSO-treated ocular surface. Data are shown as the mean ± S.D. of three (a and b) or four (c, d, and e) independent experiments. $*P < 0.05$; $**P < 0.01$; n.s., no significance.
